# Supplementary material for: Structural insights into the iron nitrogenase complex
Source: Nat Struct Mol Biol. 2023 Dec 7;31(1):150–8. doi: 10.1038/s41594-023-01124-2 (PMC10803253; doi:10.1038/s41594-023-01124-2)
Supplement: Supplementary file 1 — Supplementary Tables 1–3 [file 41594_2023_1124_MOESM1_ESM.pdf]

nature structural & molecular biology

Article https://doi.org/10.1038/s41594-023-01124-2

Structural insights into the iron nitrogenase complex

In the format provided by the authors and unedited

Supplementary Information

Table S1: Primers used in this study.

Primer	Target	Sequence (5´-3´)	Purpose
oMM0021	B10S genomic DNA	GCAGCGTGAAGCAGCCCGTTCGGAATTCCG	Construction of pMM0064
oMM0023	pRhon5Hi-2	CTGCTTCACGCTGCCGCAAG	Construction of pMM0064
oMM0027	pBS85	GTTTTTGGTCTCAGCCAATCCCTGGG	Construction of pMM0021
oMM0028	pBS85	GGCTGAGACCAAAAACATATTCTCAATAAACCC	Construction of pMM0021
oMM0033	pOGG024	CACCACAGGTCTCGGGGTTATGCAGCGGAAAAGG	Construction of pMM0002
oMM0034	pOGG024	TCAGTAGGTCTCGAGCATGGTGAGAATCCAGGGGTCC	Construction of pMM0002
oMM0035	B10S genomic DNA	TACAACAGGTCTCGTGCTCCCGAGGCGAC	Construction of pMM0002
oMM0036	B10S genomic DNA	CATCATGGTCTCGACCGGGTAAGGAGTTCCTGTC	Construction of pMM0002
oMM0037	B10S genomic DNA	CACCACAGGTCTCGACCCATTCCAAGGGCCG	Construction of pMM0002
oMM0038	B10S genomic DNA	CAATCAGGTCTCGTGGCTGCAGATCCAGTCCGTA	Construction of pMM0002
oMM0054	B10S genomic DNA	CGTAGTCTGACGATGCGCACTTC	Construction of pMM0057
oMM0055	B10S genomic DNA	GTCAAAGGAGGCAAGCCCCATCACGAG	Construction of pMM0057
oMM0056	B10S genomic DNA	GGGCTTGCCTCCTTTGACTTGCTCGGGTT	Construction of pMM0057
oMM0057	B10S genomic DNA	GGATCCTCTAGTGCAGAACCGAATCCGAAAGC	Construction of pMM0057
oMM0145	pRhon5Hi-2	ATCGCCGCGCAACCTGGATCCGAATTCGAGCTCCGTCGACAAGCTTG	Construction of pMM0064
oMM0146	B10S genomic DNA	CTCGAATTCGGATCCAGGTTGCGCGGCGATG	Construction of pMM0064

oMM0161	pMM0064	AAAGCGGTGTCCGAGATGACCGGCCAGC	Construction of pMM0073
oMM0162	pMM0064	CCGAGATCTTCGGGAGCGCCTGAAGC	Construction of pMM0073
oMM0163	pMM0064	CATCTCGGACACCGCTTTCAGGAAGGC	Construction of pMM0073
oMM0164	pMM0064	CCGAAGATCTCGGGCCGTCTCTTGGGC	Construction of pMM0073
oMM0223	pMM0119	CGTTCAACCTGCCGCCGAATGGAGCCACCCGCAGTTCGAAAAAT	Construction of pMM0190
oMM0224	pMM0119	TTTCGCTGATATCGGTCATCTGTCCCTTATTTTTCGAACTGCGGGTGGCT	Construction of pMM0190
oMM0227	pK18mobSacB	GATTTAGGTCTCTGTAAAACGACGGCCAGTGC	pK18mobSacB backbone amplification for Gibson assembly
oMM0228	pK18mobSacB	GATTTAGGTCTCTCGTAATAGCGAAGAGGCCCG	pK18mobSacB backbone amplification for Gibson assembly
oMM0284	B10S genomic DNA	GATTTAGGTCTCTTTACGCGCGACATCATCTTCGG	Construction of pMM0105
oMM0286	B10S genomic DNA	GATTTAGGTCTCAGAGATCCGCGCTCAGGTGC	Construction of pMM0105
oMM0287	B10S genomic DNA	GATTTAGGTCTCTTACGAACGAATATCTGGCGGCGG	Construction of pMM0105
oMM0288	B10S genomic DNA	GATTTAGGTCTCTTCTCCAGATTGGTGGAATGGCCAAGG	Construction of pMM0105
oMM0323	B10S genomic DNA	ATGCAAGCTTGGCACTGGCCGTCGTTTTACGGTCCTCGCAGCGGTC	Construction of pMM0133
oMM0324	B10S genomic DNA	CCCTAGCCCCCCCTGGAGACGAACACGCGCGA	Construction of pMM0133
oMM0325	B10S genomic DNA	GCGCGTGTTCGTCTCCAGGGGGGGCTAGGGTG	Construction of pMM0133

oMM0326	B10S genomic DNA	GCGATCGGTGCGGGCCTCTTCGCTATTACGCTCGGGTTTTCGGTGGTGG	Construction of pMM0133
oMM0384	pRhon5Hi-2	GCCGTGGGTCGATGTTTGATGTTAGTCTTATCTGAAAGTTGTGC	Construction of pMM0119
oMM0385	pRhon5Hi-2	CGATCTCGGCTTGAACGAATTGACCTTTTCTCCGACGAATAGAGT	Construction of pMM0119
oMM0386	pOGG024	AATTCGTTCAAGCCGAGATCG	Construction of pMM0119
oMM0387	pOGG024	TAACATCAAACATCGACCCACG	Construction of pMM0119
oMM0389	pMM0073	GATTTAGGTCTCAGGAGCAGCCCGTTCGGAATTCC	Construction of pMM0119
oMM0390	pMM0073	GATTTAGGTCTCTAGCGGGTCACCACACGTTGAGG	Construction of pMM0119
oMM0394	B10S genomic DNA	GATTTAGGTCTCTCTGGTCAAGATCCTCGACGC	Construction of pMM0131
oMM0395	B10S genomic DNA	GATTTAGGTCTCTCCAGCGACTTGCCGATACCACCTTTG	Construction of pMM0131
oMM0396	B10S genomic DNA	GATTTAGGTCTCAGGAGATCCCGATCCCGTTCGAATG	Construction of pMM0131
oMM0397	B10S genomic DNA	CAAATCGACGACCACCATGGCCAACACGCTTTTCG	Construction of pMM0131
oMM0510	pMM0119	CCTTCCCAAGGAGCGACCCATGCACCACCATCATCACCATA	Construction of pMM0190
oMM0511	pMM0119	CCGTAAATGGCGATCTTGCGGGTATGGTGATGATGGTGGTGC	Construction of pMM0190

Table S2: Plasmids used in this study.

Plasmid	Code	Relevant Features	Source or Reference
pK18mobSacB	pMM0056	Suicide vector, *oriT* (mobilizable), *sacB,* Kan^R	[1]
pK18mobSacB-*draTG*	pMM0105	Suicide vector, *oriT* (mobilizable), *sacB,* Kan^R, homologous recombination sites for draTG locus	This study
pK18mobSacB-*gtaI*	pMM0133	Suicide vector, *oriT* (mobilizable), *sacB,* Kan^R, homologous recombination sites for gtaI locus	This study
pK18mobSacB-*nifHDK*	pMM0131	Suicide vector, *oriT* (mobilizable), *sacB,* Kan^R, homologous recombination sites for nifHDK locus	This study
pK18mobSacB-*modABC*	pMM0057	Suicide vector, *oriT* (mobilizable), *sacB,* Kan^R, homologous recombination sites for modABC locus	This study
pBS85	pMM0017	Suicide vector, *oriT* (mobilizable), Tet^R	[2]
pBS85-BsaI	pMM0021	Suicide vector, *oriT* (mobilizable), Tet^R, BsaI cutting site	This study
pBS85-BsaI-*genR*	pMM0002	Suicide vector, *oriT* (mobilizable), Tet^R, BsaI cutting site, Gen^R, homologous recombination sites for anfHDGK locus	This study
pRhon5Hi-2	pMM0031	Broad host range plasmid, Kan^R	[3]
pRhon5Hi-2-*anfHDGK*	pMM0064	Broad host range plasmid, *anfHDGK* operon, Kan^R	This study
pRhon5Hi-2-*anfHDGK*-Golden Gate	pMM0073	Broad host range plasmid, *anfHDGK* operon with silent mutation in BsaI cutting site, Kan^R	This study
pOGG024	pMM0114	Broad host range plasmid, *oriT* (mobilizable)*, lacZα* cassette for golden gate cloning (BsaI), Gen^R	Addgene plasmid #113991
pOGG024-*kanR*	pMM0119	Broad host range plasmid, *oriT* (mobilizable)*, lacZα* cassette for golden gate cloning (BsaI), Kan^R	This study
pOGG024-*kanR*-*anfHDGK*	pMM0119	Broad host range plasmid, *oriT* (mobilizable)*, anfHDGK* operon, Kan^R	This study
pOGG024-*kanR*-*anfHDGK*-Strep/His	pMM0190	Broad host range plasmid, *oriT* (mobilizable)*, anfHDGK* operon with N-terminal His$_6$-tag and C-terminal Strep tag II, Kan^R	This study

Table S3: Strains used in this study.

Strain	Genotype	Source or Reference
Rhodobacter capsulatus B10S	Wildtype	[4]
Rhodobacter capsulatus MM0425	Δ*anfHDGK*::*genR* Δ*modABC* Δ*draTG* Δ*gtaI* Δ*nifHDK*	This study
Rhodobacter capsulatus MM0436 (expression strain)	Δ*anfHDGK*::*genR* Δ*modABC* Δ*draTG* Δ*gtaI* Δ*nifHDK*	This study
Escherichia coli DH5α	F^- Φ80*lac*ZΔM15 Δ(*lac*ZYA-*arg*F) U169 *rec*A1 *end*A1 *hsd*R17(r_k^-, m_k^+) *pho*A *sup*E44 *thi*-1 *gyr*A96 *rel*A1 λ^-	Thermo Fisher Scientific Inc. (Waltham, USA) catalogue #18265017
Escherichia coli ST18	RP4-2 *Tc*::*Mu* *Km*::*Tn7* Δ*hemA* mutant	[5]

SI References

1. Schäfer, A., et al., *Small mobilizable multi-purpose cloning vectors derived from the Escherichia coli plasmids pK18 and pK19: selection of defined deletions in the chromosome of Corynebacterium glutamicum.* Gene, 1994. **145**(1): p. 69-73.
2. Hoffmann, M.C., et al., *Coordinated expression of fdxD and molybdenum nitrogenase genes promotes nitrogen fixation by Rhodobacter capsulatus in the presence of oxygen.* Journal of Bacteriology, 2014. **196**(3): p. 633-40.
3. Troost, K., et al., *Engineered Rhodobacter capsulatus as a Phototrophic Platform Organism for the Synthesis of Plant Sesquiterpenoids.* Frontiers in Microbiology, 2019. **10**: p. 1998.
4. Klipp, W., B. Masepohl, and A. Pühler, *Identification and mapping of nitrogen fixation genes of Rhodobacter capsulatus: duplication of a nifA-nifB region.* Journal of Bacteriology, 1988. **170**(2): p. 693-699.
5. Thoma, S. and M. Schobert, *An improved Escherichia coli donor strain for diparental mating.* FEMS Microbiology Letters, 2009. **294**(2): p. 127-132.
