# Supplementary material for: Structural insights into the iron nitrogenase complex
Source: Nat Struct Mol Biol. 2023 Dec 7;31(1):150–8. doi: 10.1038/s41594-023-01124-2 (PMC10803253; doi:10.1038/s41594-023-01124-2)
Supplement: Supplementary file 2 — Reporting Summary [file 41594_2023_1124_MOESM2_ESM.pdf]

nature portfolio

Corresponding author(s): Johannes G Rebelein

Last updated by author(s): Aug 9, 2023

Reporting Summary

Nature Portfolio wishes to improve the reproducibility of the work that we publish. This form provides structure for consistency and transparency in reporting. For further information on Nature Portfolio policies, see our Editorial Policies and the Editorial Policy Checklist.

Statistics

For all statistical analyses, confirm that the following items are present in the figure legend, table legend, main text, or Methods section.

n/a	Confirmed	
☐	☒	The exact sample size (*n*) for each experimental group/condition, given as a discrete number and unit of measurement
☐	☒	A statement on whether measurements were taken from distinct samples or whether the same sample was measured repeatedly
☒	☐	The statistical test(s) used AND whether they are one- or two-sided<br>*Only common tests should be described solely by name; describe more complex techniques in the Methods section.*
☒	☐	A description of all covariates tested
☒	☐	A description of any assumptions or corrections, such as tests of normality and adjustment for multiple comparisons
☐	☒	A full description of the statistical parameters including central tendency (e.g. means) or other basic estimates (e.g. regression coefficient) AND variation (e.g. standard deviation) or associated estimates of uncertainty (e.g. confidence intervals)
☒	☐	For null hypothesis testing, the test statistic (e.g. *F*, *t*, *r*) with confidence intervals, effect sizes, degrees of freedom and *P* value noted<br>*Give P values as exact values whenever suitable.*
☒	☐	For Bayesian analysis, information on the choice of priors and Markov chain Monte Carlo settings
☒	☐	For hierarchical and complex designs, identification of the appropriate level for tests and full reporting of outcomes
☒	☐	Estimates of effect sizes (e.g. Cohen's *d*, Pearson's *r*), indicating how they were calculated

Our web collection on statistics for biologists contains articles on many of the points above.

Software and code

Policy information about availability of computer code

Data collection	TotalChrom v. 6.3.4 (PerkinElmer), AcquireMP v. 2.3 (Refeyn Ltd), ICP Expert Software v. 4.1.0 (Agilent), EPU software v. 2.9 - 2.11 (Thermo Scientific)
Data analysis	GraphPad Prism 9, DiscoverMP v. 2022 R1 (Refyn Ltd), CryoSPARC 4.1, ChimeraX 1.5, Chimera 1.16, PHENIX 1.21.1, Pymol 2.5, COOT 0.8.9.2, CAVER 3.0.3, AlphaFold 2 (Google DeepMind)

For manuscripts utilizing custom algorithms or software that are central to the research but not yet described in published literature, software must be made available to editors and reviewers. We strongly encourage code deposition in a community repository (e.g. GitHub). See the Nature Portfolio guidelines for submitting code & software for further information.

Data

Policy information about availability of data

All manuscripts must include a data availability statement. This statement should provide the following information, where applicable:

- Accession codes, unique identifiers, or web links for publicly available datasets
- A description of any restrictions on data availability
- For clinical datasets or third party data, please ensure that the statement adheres to our policy

All unique materials used in this study are available from the corresponding author upon request. All raw data for mass photometry measurements, kinetic experiments, and protein characterisation are deposited on Edmond, the Open Research Data Repository of the Max Planck Society for public access (DOI: https://

doi.org/10.17617/3.P6SEVC) [67]. The structures reported in this paper are deposited to the Protein Data Bank under the accession codes 8OIE (ADP-AlF3-stabilized Fe nitrogenase complex) and 8PBB (CHAPSO treated partial Fe nitrogenase catalytic component). CryoEM data were deposited to the Electron Microscopy Data Bank under EMD-16890 (ADP-AlF3-stabilized Fe nitrogenase complex) and EMD-17583 (CHAPSO treated partial Fe nitrogenase catalytic component).

The structures used to build our model and compare our structure to are available at the Protein Data Bank under identifiers: 7QQA, 7UTA, 5N6Y. Sequences for the protein alignment were obtained from NCBI non-redundant protein database and are available as table in the source data on EDMOND (DOI: https://doi.org/10.17617/3.P6SEVC).

Research involving human participants, their data, or biological material

Policy information about studies with human participants or human data. See also policy information about sex, gender (identity/presentation), and sexual orientation and race, ethnicity and racism.

Reporting on sex and gender	The research did not involve human participants, their data, or biological material.
Reporting on race, ethnicity, or other socially relevant groupings	The research did not involve human participants, their data, or biological material.
Population characteristics	The research did not involve human participants, their data, or biological material.
Recruitment	The research did not involve human participants, their data, or biological material.
Ethics oversight	The research did not involve human participants, their data, or biological material.

Note that full information on the approval of the study protocol must also be provided in the manuscript.

Field-specific reporting

Please select the one below that is the best fit for your research. If you are not sure, read the appropriate sections before making your selection.

☒ Life sciences ☐ Behavioural & social sciences ☐ Ecological, evolutionary & environmental sciences

For a reference copy of the document with all sections, see nature.com/documents/nr-reporting-summary-flat.pdf

Life sciences study design

All studies must disclose on these points even when the disclosure is negative.

Sample size	A sample size of n=3 was used for most experiments according to standard scientific practice to validate data quality, repeatability of experiments and reliability of measurement devices. For ICP-OES measurements 2 technical replicates of 2 biological replicates were used. This combination of biological and technical replicates was chosen to ensure robustness and reproducibility of the assay, as well as to validate results on independent days.
Data exclusions	No data was excluded.
Replication	Most experiments were performed as replicates (n=3) to ensure repeatability and reliability of measurement devices. The number of replications is given in the figure legend for individual experiments and, where applicable, shown as individual data points. Only ICP-OES experiments were performed as a combination of technical and biological replicates to ensure reproducibility on independent days. Single particle cryoEM is based on an average of protein particles within a vitreous layer of ice. Replication is therefore not necessarily required to ensure statistical robustness of structural data. In this work we determined the structure of the iron nitrogenase with and without reductase component with good overlap of core structural features, which can, in essence, be regarded as a biological replicate.
Randomization	No randomization was required for the experimental design and workflow of this study. It is of note that stochasticity is introduced during individual data processing steps in CryoSPARC 4.1 (e.g., during 2D classification and during randomization of half map sets during 3D refinement). In the final reconstruction, randomized half sets of particles are used to determine gold-standard Fourier shell correlations at 0.143 level.
Blinding	For all studies in this manuscript there was no awareness of group assignment that would have caused biased results. Therefore, blinding was not relevant for data reliability.

Reporting for specific materials, systems and methods

We require information from authors about some types of materials, experimental systems and methods used in many studies. Here, indicate whether each material, system or method listed is relevant to your study. If you are not sure if a list item applies to your research, read the appropriate section before selecting a response.

Materials & experimental systems

n/a	Involved in the study
☒	☐ Antibodies
☒	☐ Eukaryotic cell lines
☒	☐ Palaeontology and archaeology
☒	☐ Animals and other organisms
☒	☐ Clinical data
☒	☐ Dual use research of concern
☒	☐ Plants

Methods

n/a	Involved in the study
☒	☐ ChIP-seq
☒	☐ Flow cytometry
☒	☐ MRI-based neuroimaging
