# Supplementary figures and images for: Structural insights into the iron nitrogenase complex
Source: Nat Struct Mol Biol. 2023 Dec 7;31(1):150–8. doi: 10.1038/s41594-023-01124-2 (PMC10803253; doi:10.1038/s41594-023-01124-2)

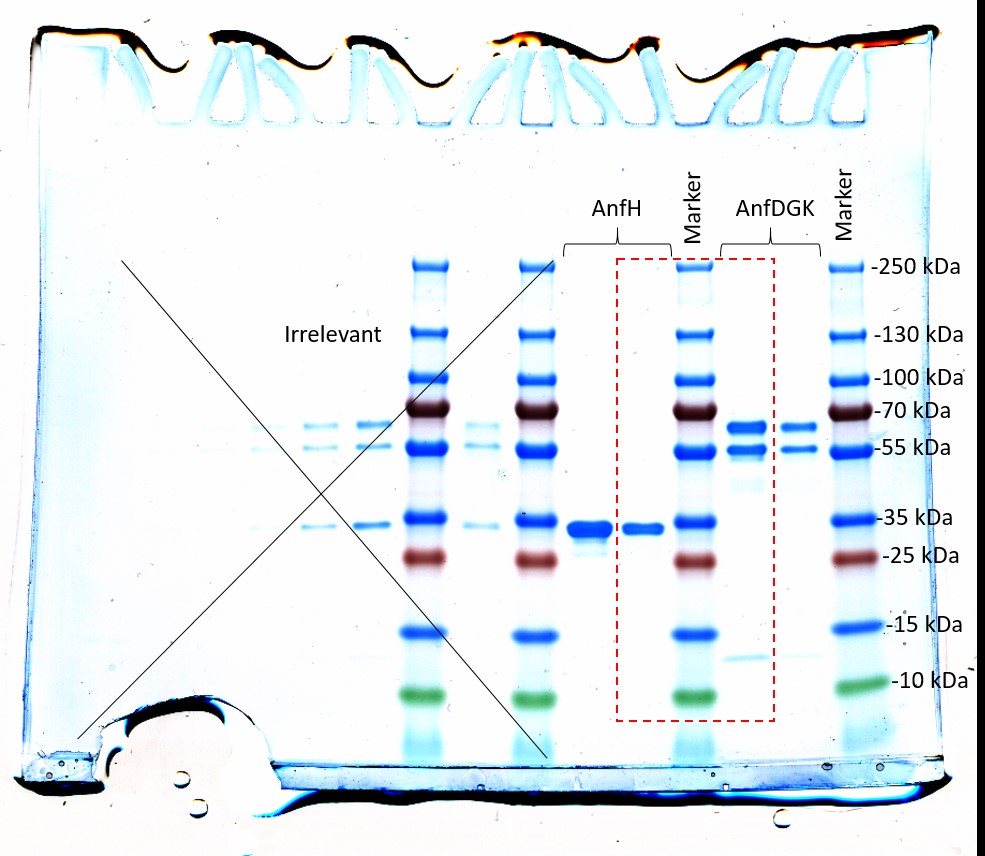

Supplement: Supplementary file 6 — Unprocessed gel. [file 41594_2023_1124_MOESM6_ESM.jpg]
